# Prognostic Impact of Pulmonary Diseases in 952 Patients with Thoracic and/or Abdominal Aortic Aneurysm

**DOI:** 10.3390/jcm13206247

**Published:** 2024-10-19

**Authors:** Yoichi Kobayashi, Takashi Ishiguro, Naho Kagiyama, Makoto Sumi, Noboru Takayanagi

**Affiliations:** 1 Department of Respiratory Medicine, Saitama Cardiovascular and Respiratory Center, 1696 Itai, Kumagaya 360-0197, Saitama, Japan; 2 Department of Vascular Surgery, International University of Health and Welfare, 2600-1 Kita-Kanemaru, Ohtawara 324-8501, Tochigi, Japan

**Keywords:** acute exacerbation, aortic aneurysm, idiopathic pulmonary fibrosis, lung cancer, pulmonary disease

## Abstract

**Background/Objectives:** Pulmonary diseases are common in patients with thoracic aortic aneurysm (TAA) and abdominal aortic aneurysm (AAA). Although high prevalences of chronic obstructive pulmonary disease and lung cancer (LC) are known, the prevalence of these and other pulmonary diseases regarding their relation to the outcome of TAA and/or AAA are not determined. **Methods:** Pulmonary diseases present at aortic aneurysm (AA) diagnosis and follow-up periods and cause of death of 952 patients with TAA, AAA, or TAA + AAA (including thoracoabdominal AA) treated at our institution in Japan were retrospectively analyzed. Cox regression analysis was used to investigate potential risk factors of mortality. **Results:** The mean patient age was 72.4 years, and the median follow-up was 4.92 years. At diagnosis, 528 (55.5%) patients had pulmonary diseases, including emphysema without interstitial lung disease (ILD) or LC, LC, idiopathic pulmonary fibrosis (IPF) without LC, non-IPF ILD without LC, and interstitial lung abnormalities (ILAs) without LC in 250, 85, 65, 15, and 58 patients, respectively. During follow-up, LC and acute exacerbation (AE) of IPF developed in 50 and 12 patients, respectively. In 213 patients who died, there were 45 (21.1%) aortic disease-related deaths. Other causes of death included LC (27.7%), cardiovascular events (9.4%), pneumonia (5.6%), and interstitial lung disease (4.7%). In a multivariate Cox regression hazard model, age; larger maximum aneurysm diameter; and coexisting LC, IPF, or concomitant cancer were associated with poor prognosis. **Conclusions:** In patients with AA, not only age and aneurysm diameter but also coexisting LC and IPF were prognostic factors for mortality.

## 1. Introduction

Aortic aneurysm (AA) remains a disease with a high mortality rate when it ruptures [1,2,3,4,5]. The need for prophylactic surgical or endovascular repair is based on aneurysm size, growth, and other associated conditions. The decision to repair an AA requires consideration of balancing risks with an individual patient’s probable life expectancy [6]. Thus, it is clinically important to elucidate the prognostic factors of all-cause mortality at AA diagnosis.

AA and pulmonary disease are closely related as both diseases are associated with smoking [2,7,8]. Pulmonologists are often consulted about pulmonary diseases by vascular surgeons at AA diagnosis, at AA repair, and during follow-up periods. Pulmonary disease, particularly chronic obstructive pulmonary disease (COPD), has been reported to have a poor prognosis in AA patients [9,10,11], and several studies have documented the high risk of AA patients for developing lung cancer (LC) [12,13]. In contrast, there are few reports on the association between AA and idiopathic pulmonary fibrosis (IPF), non-IPF interstitial lung disease (ILD), and interstitial lung abnormality (ILA) [14]. Furthermore, data on the incidence and treatment of LC are limited. The identification and prompt treatment of pulmonary diseases, especially LC, may have a clinically significant and meaningful effect on overall outcomes for patients with AA.

In this retrospective study, we, as pulmonologists, hypothesized that the survival of AA patients with IPF, emphysema, or LC at diagnosis would be shorter than in patients without these pulmonary diseases. We evaluated pulmonary diseases at AA diagnosis and during follow-up, the incidence density of LC and acute exacerbation (AE) of IPF development during follow-up, and staging and therapy of LC of AA patients. Then, we reviewed the causes of death to elucidate the importance of these pulmonary diseases. Finally, we evaluated risk factors for mortality, especially of pulmonary diseases, in AA patients at AA diagnosis.

## 2. Materials and Methods

### 2.1. Subjects

From August 2003 through April 2020, 1056 patients were diagnosed as having thoracic aortic aneurysm (TAA), abdominal aortic aneurysm (AAA), or both, including thoracoabdominal aortic aneurysm (TAAA), at our institution. Of these patients, 44 were excluded because AA was ruptured at diagnosis and 60 due to an observation period of less than 3 months. The remaining 952 patients comprised the study cohort.

### 2.2. Definitions

AA was diagnosed according to the guidelines of the Japanese Circulation Society [15]. IPF was diagnosed according to the official American Thoracic Society/European Respiratory Society/Japanese Respiratory Society/Latin American Thoracic Society statement on IPF [16]. AE-IPF was defined based on clinical and radiological features that included acute onset of dyspnea less than one month in duration, bilateral changes in ground-glass opacities on chest high-resolution computed tomography (HRCT), and absence of an identifiable etiology [17]. Emphysema was considered present if low-attenuation areas were present on HRCT images. Non-IPF ILD was diagnosed according to each diagnostic criterion. ILA was diagnosed when incidental non-dependent abnormalities, including ground-glass or reticular abnormalities, lung distortion, traction bronchiectasis, honeycombing, and non-emphysematous cysts involving at least 5% of a lung zone, were identified [18]. LC was basically diagnosed by pathology and cytology, but some patients were diagnosed clinically and radiologically because bronchoscopy was considered risky. When cytological findings suggested non-small cell LC but histological type was not specified, LC was diagnosed as unclassified non-small cell LC. In patients with LC and IPF, non-IPF ILD, ILA, or emphysema, the diagnosis was LC in the present study. Similarly, in patients with IPF, non-IPF ILD, or ILA and emphysema, the diagnosis was IPF, non-IPF ILD, or ILA. So, a diagnosis of emphysema was made in patients who only had emphysema. Some patients had both TAA and AAA or just TAAA. These patients were classified as having TAA + AAA. Day zero was defined as the patient’s first visit to our hospital when patients were referred to a vascular surgeon or to The Department of Respiratory Medicine for pulmonary disease and then an AA was discovered. This study was approved by the institutional review board of Saitama Cardiovascular and Respiratory Center (No. 2021039), which waived the necessity to obtain informed consent.

### 2.3. Study Design

This was a retrospective cohort study. We investigated the prevalence of pulmonary diseases at AA diagnosis, including IPF; non-IPF ILD, such as collagen vascular disease-associated ILD, non-IPF idiopathic interstitial pneumonia, fibrosing hypersensitivity pneumonitis, and sarcoidosis; ILAs; pulmonary infections, such as acute pneumonia, tuberculosis, non-tuberculosis mycobacterial infection, and chronic pulmonary aspergillosis; LC; and emphysema. We also investigated the diameter and shape of the aneurysm and systemic complications at the time of AA diagnosis. Data and outcomes were collected from the medical records. During follow-up periods, we investigated the incidence of the abovementioned pulmonary diseases and AE-IPF. We also examined causes of death and survival.

### 2.4. Statistical Analysis

Categorical baseline characteristics are summarized by frequency and percent, and continuous characteristics are reported as the mean ± standard deviation or median and interquartile range as appropriate. Group comparisons were made using a Wilcoxon rank-sum test, Pearson’s Chi-squared test, or Kruskal–Wallis rank sum test as appropriate. Diagnosis of LC and AE-IPF was estimated by Kaplan–Meier analysis. Survival was evaluated using a Kaplan–Meier curve and compared between groups using log-rank tests. Cox proportional hazards regression analysis was used to determine whether the factors at diagnosis increased the risk of all-cause mortality. In all analyses, *p* < 0.05 was considered to be statistically significant. We conducted all statistical analyses with R version 4.2.1 (R Foundation for Statistical Computing).

## 3. Results

### 3.1. Patient Characteristics by Aneurysm Type

Of the 952 patients with a mean age of 72.4 years, 781 (82.0%) patients were male, and 218 (22.9%), 650 (68.3%), and 84 (8.8%) patients were diagnosed as having TAA, AAA, and TAA + AAA, respectively. Among them, 781, 454, 111, and 171 patients had hypertension, dyslipidemia, valvular heart disease, and cerebrovascular disease, respectively. A total of 57 (6.0%) patients had concomitant cancer except for LC at AA diagnosis, including prostate cancer in 20, gastric cancer in 8, colorectal cancer in 8, bladder cancer in 7, and other cancers in 14. The mean maximum AA diameter was 48 mm overall. Dyslipidemia and coronary heart disease occurred significantly less frequently in TAA patients than in AAA or TAA + AAA patients. However, valvular heart disease occurred significantly more frequently in TAA patients than in AAA or TAA + AAA patients (Table 1).

### 3.2. Pulmonary Diseases at AA Diagnosis

Of the 952 patients, 528 (55.5%) had pulmonary diseases at AA diagnosis, including IPF in 65, non-IPF ILD in 15, ILA in 58, emphysema in 250, LC in 85, and other lung diseases in 55. Emphysema occurred significantly less frequently in TAA patients than in AAA or TAA + AAA patients (Table 1). Among all patients, 858 (90.1%) were referred to a vascular surgeon of our hospital, and 94 were referred to the Department of Respiratory Medicine for pulmonary disease and then AA was discovered. Of the 858 patients referred to a vascular surgeon, 439 (51.2%) patients had pulmonary disease at AA diagnosis and 19 (2.2%) were diagnosed as having concomitant LC.

### 3.3. Pulmonary Diseases during Follow-Up Periods

Of the 952 patients, 126 (13.2%) developed pulmonary diseases over a median follow-up period of 4.92 years (interquartile range: 2.40–8.13 years). Of these patients, 50 developed acute pneumonia, 50 developed LC, 9 developed chronic pulmonary infections (4 with non-tuberculous mycobacterial infection, 3 with tuberculosis, and 2 with chronic pulmonary aspergillosis), and 12 developed AE-IPF (Table 2).

### 3.4. LC at AA Diagnosis/Follow-Up

**Figure 1:**
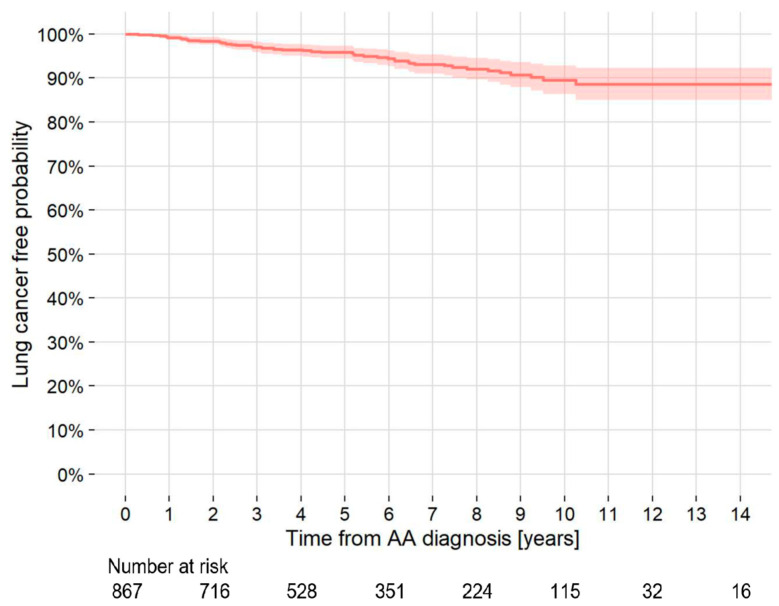
Kaplan–Meier curve of the probability of development of lung cancer in patients with TAA and/or AAA who did not have lung cancer at the AA diagnosis. AAA = abdominal aortic aneurysm; TAA = thoracic aortic aneurysm; AA = aortic aneurysm.

The 5- and 10-year cumulative rates of LC development were 4.4% and 11.0%, respectively. The incidence density of LC development was 10.5 cases/1000 person-years (Figure 1). Of the 85 patients with LC at AA diagnosis, 31 (36.5%) were stage I, and of the 50 patients who developed LC during follow-up, 28 (56.0%) were stage I. The number of stage I patients with LC at AA diagnosis tended to be lower than that with LC during follow-up, although the difference was not significant (*p* = 0.15) (Table 3).

### 3.5. AE-IPF

**Figure 2:**
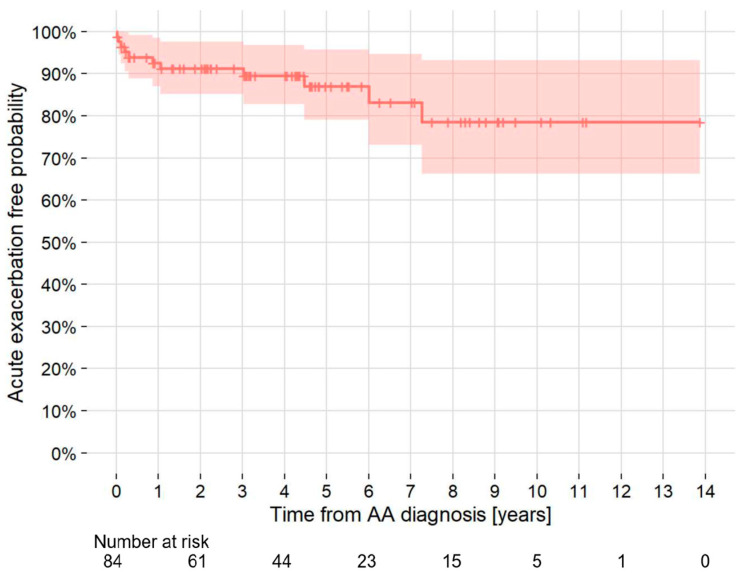
Kaplan–Meier curve of the probability of development of acute exacerbation of idiopathic pulmonary fibrosis in patients with TAA and/or AAA who had idiopathic pulmonary fibrosis at AA diagnosis. AA = aortic aneurysm; AAA = abdominal aortic aneurysm; TAA = thoracic aortic aneurysm.

Of the 84 patients diagnosed as having IPF with or without LC at the time of AA diagnosis, including 19 patients with concomitant LC and IPF, 12 (14.3%) patients developed AE-IPF during follow-up. The 5- and 10-year cumulative rates of AE-IPF development were 13.0% and 21.4%, respectively. The incidence density of AE-IPF development in all patients was 30.5 cases/1000 person-years (Figure 2). Two patients developed AE-IPF within one month after thoracic endovascular aortic repair (TEVAR) or open surgery. Of the 54 patients with IPF who underwent TEVAR or endovascular aortic repair (EVAR), one (1.9%) patient developed AE-IPF within one month after TEVAR. Of the two patients with IPF who underwent open surgery, one (50.0%) patient developed AE-IPF within one month after open surgery. Of the 14 patients with IPF who underwent LC surgery, 2 (14.3%) patients developed AE-IPF within one month after surgery.

### 3.6. Open or Endovascular Repair of TAA, TAAA, or AAA

Of the 952 AA patients, 226 underwent no repair of AA, 570 received endovascular repair of AA, and 156 received surgical repair of AA. The 6-month and 1-year cumulative repair rates were 51.1% and 56.8%, respectively. Of the 218 TAA patients, 61 underwent no repair of TAA, 128 received TEVAR, and 74 received surgical repair. Of the 650 AAA patients, 148 underwent no repair of AAA, 431 received EVAR, and 71 received surgical repair. Of the 84 TAA + AAA patients, 17 underwent no repair of TAA + AAA, 56 received TEVAR or EVAR, and 11 received surgical repair. All treatment strategies were selected by expert consensus.

### 3.7. Mortality by Location and Disease

**Figure 3:**
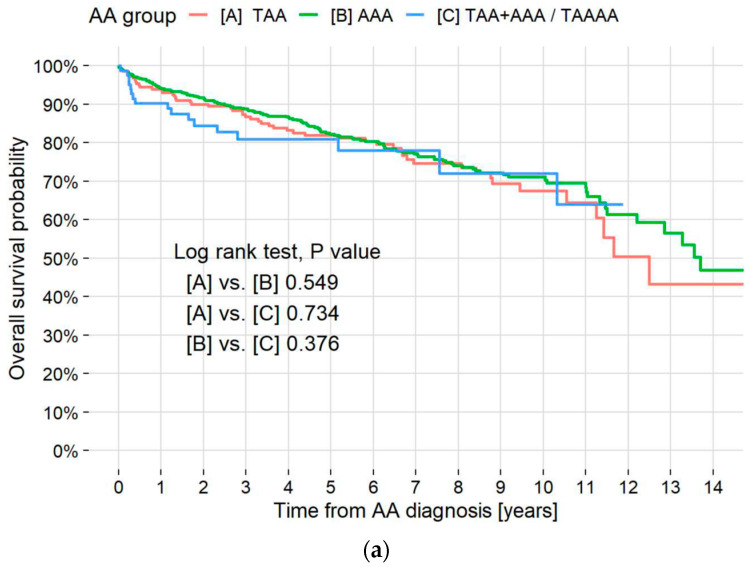

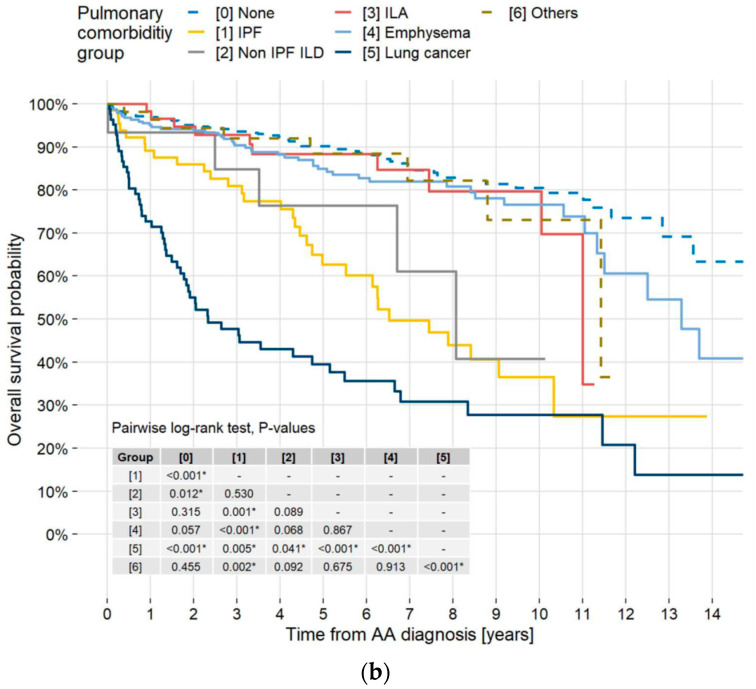
Kaplan–Meier survival curves by aneurysm type and pulmonary disease. (**a**) Kaplan–Meier survival curves of all-cause mortality in all patients and those with TAA, AAA, and TAA + AAA. Overall cumulative 5- and 10-year mortality rates were 18.0% and 29.8%, respectively. Respective 5- and 10-year all-cause mortality rates in the TAA, AAA, and TAA + AAA patients were 18.1% and 32.4%, 17.7% and 28.9%, and 19.0% and 27.9%. A log-rank test showed no significant difference between survival curves in the patients with TAA, AAA, and TAA + AAA. Median survival time for all AA patients was 13.6 years (95% CI, 12.5–NR). (**b**) Kaplan–Meier survival curves of all-cause mortality in patients without pulmonary disease and those with IPF, non-IPF ILD, ILAs, emphysema, LC, and others. Respective 5-year all-cause mortality rates in the patients without pulmonary disease, patients with IPF, non-IPF ILD, ILAs, emphysema, LC, and others were 9.8%, 37.4%, 23.6%, 11.6%, 15.0%, 60.5%, and 11.5%. A pairwise log-rank test showed a significant difference between patients without pulmonary diseases and patients with LC, IPF, and non-IPF ILD (*p* <0.001, <0.001, and =0.012, respectively). * in the table indicates statistical significance. TAA = thoracic aortic aneurysm; AAA = abdominal aortic aneurysm; AA = aortic aneurysm; CI = confidence interval; NR = not reached; IPF = idiopathic pulmonary fibrosis; ILD = interstitial lung disease; ILAs = interstitial lung abnormalities; LC = lung cancer.

Death from any cause occurred in 213 (22.4%) patients, and median survival times in years of all patients and patients with TAA and AAA were 13.6, 12.5, and 13.7, respectively. The survival rate of the patients with TAA + AAA was higher than 50% at the end of the study (Figure 3a). Five-year survival rates of patients without pulmonary diseases and patients with LC, IPF, non-IPF ILD, ILA, emphysema, and other pulmonary diseases were 90.2%, 39.5%, 62.6%, 76.4%, 88.4%, 85.0%, and 88.5%, respectively (Figure 3b). The log-rank test or pairwise log-rank test showed no significant difference between TAA, AAA, and TAA + AAA survival curves (*p* = 0.549, 0.734, and 0.376, respectively) but did show a significant difference between patients without pulmonary diseases and those with LC, IPF, and non-IPF ILD (*p* < 0.001, <0.001, =0.012, respectively).

### 3.8. Causes of Death/Risk Factors

Causes of death were aortic diseases (45 patients, 21.1%), including aortic dissection (6 patients, 2.8%), AAA rupture (17 patients, 8.0%), TAA rupture (20 patients, 9.4%), and vascular graft infection (2 patients, 0.9%); LC (59 patients, 27.7%); cardiovascular events (20 patients, 9.4%); other malignancy (18 patients, 8.5%); pneumonia (12 patients, 5.6%); and interstitial lung disease (10 patients, 4.7%). Respiratory diseases accounted for 83 of the 213 deaths (39.0%) (Table 4).

A univariate Cox proportional hazard model showed age, AA diameter, the presence of LC, IPF, and concomitant cancer to be negative prognostic factors, whereas the presence of hypertension, coronary artery disease, and dyslipidemia was a positive prognostic factor of death (Table 5). A multivariate Cox proportional hazard model showed age, AA diameter, and presence of LC, IPF, or concomitant cancer to be negative prognostic factors and hypertension, dyslipidemia, valvular heart disease, and cerebrovascular disease to be positive prognostic factors for death (Table 5).

## 4. Discussion

Two important clinical observations were revealed in the present study. First, respiratory diseases are common in AA patients, and second, respiratory diseases are associated with poor outcomes in patients with AA.

Among the study patients, 55.5% had pulmonary diseases at AA diagnosis. Previous meta-analyses of AAA have reported rates of pulmonary disease at AA diagnosis ranging from 23.0% to 29.4% [19,20,21]. For TAA, the rate was 39.3–43.0%, although not in the meta-analyses [22,23]. Respiratory complications were more common in the present study than noted in these reports. In many of these reports, however, pulmonary disease refers mainly to COPD, whereas that in the present study includes a wide variety of diseases such as LC, ILD, chronic infections, and bronchial asthma. As only a small number of patients in the present study underwent pulmonary function tests at diagnosis, COPD could not be evaluated, and emphysema was evaluated radiologically and used instead. Thus, our findings cannot be simply compared to those of past reports. However, it should be emphasized that more than half of our patients were complicated by pulmonary disease at AA diagnosis.

Compared to control subjects, there is a higher incidence of LC complications at AAA diagnosis, as well as a higher incidence of AAA complications at LC diagnosis [13,24,25,26,27]. Cancer complications are also common in TAA. In 234 cases of TAA with descending AA, 16.7% had coexisting cancer [22]. In the present study, 8.9% had LC and 6.0% had non-LC at the AA diagnosis. This complication rate for LC is likely higher than the actual clinical frequency in AA care because it includes patients who were referred to respiratory medicine for LC and found to have AA at the same time. In the present study, LC was found in 2.2% of 858 patients referred to cardiology and vascular surgery, which would be close to the true frequency in actual AA practice.

A search for the prevalence of ILD at the AA diagnosis revealed only a few reports on this subject. A review of CT images of 98 patients with IPF revealed a reported prevalence of TAA of 6%, which is higher than that in the general population [14]. In the absence of comparable reports, the present report is clinically important, as 14.5% of our AA patients had concomitant ILD at diagnosis. Even if ILA were excluded, 8.4% of the AA patients had concomitant IPF or non-IPF ILD at diagnosis.

The present study also found that pulmonary diseases are common during AA treatment. During a median observation period of 4.92 years, pulmonary diseases occurred in 13.2% of the patients. However, no previous reports have documented the overall occurrence of pulmonary diseases during the course of AA, although there are scattered reports on LC. In one previous report, LC appeared in 12.0% of patients with AAA within 16.2 months of observation [12]. The overall incidence density of cancer, and not only of LC, was reported to be 4.36/100 person-years in patients with AAA [13]. Both reports had a higher cumulative incidence of cancer than control subjects without AA. The incidence density of LC in the present study was 10.5/1000 person-years, which is higher than the incidence density of LC in the Japanese population as a whole (1.15/1000 person-years) but lower than that of LC in patients with COPD (23.0/1000 person-years) and IPF (25.2/1000 person-years) reported in Japan to date [28,29,30]. Considering the high cumulative incidence of LC, early detection of LC by CT at the appropriate time is expected to contribute to improved patient prognosis.

IPF is a chronic, fibrosing interstitial pneumonia of unknown cause that is associated with radiological and histologic features of usual interstitial pneumonia. It is characterized by progressive worsening of dyspnea and lung function. Lung fibrosis is confidently recognized when traction bronchiectasis/bronchiolectasis and/or honeycombing are identified on CT scans [16]. AE-IPF has been defined as an acute, clinically significant respiratory deterioration of unidentifiable cause. AE-IPF is associated with high in-hospital mortality [17]. In this study, AE-IPF occurred in 21.4% of AA patients with IPF over 10 years, and the incidence density of AE-IPF was 30.5/1000 person-years. There have been no previous reports that revealed the incidence of AE-IPF in patients with AA. 

As the second important observation of the present study, respiratory diseases are associated with poor outcomes in AA patients. Respiratory diseases accounted for 83 of the 213 deaths (39.0%) and were more frequently a cause of all-cause death than AA-related disease (21.1%) and cardiovascular diseases (9.4%). Meta-analyses of the DREAM, EVAR-1, and EVAR-2 trials showed that pulmonary diseases, excluding LC, accounted for 8.4–17.1% of the deaths [20,31,32]. When LC is excluded from the causes of death in the present study, deaths due to pulmonary diseases account for 11.3%, which is comparable to previously reported rates. In the present study, LC accounted for 27.7% of deaths, a higher percentage than that for EVAR-1 (10.0%) and EVAR-2 (5.5%). However, we consider that the differences were a result of the large number of patients referred for LC in the present study.

Our multivariate analysis revealed that age, larger maximum aneurysm diameter, IPF, and LC were negative prognostic factors. This is the first report, to our knowledge, of IPF as a negative prognostic factor in a multivariate analysis of patients with AA. COPD has been reported as a poor prognostic factor in multivariate analyses [9,10,11], but in the present study, emphysema was not a prognostic factor. Many of the patients with emphysema in the present study had concomitant LC or IPF, and we omitted cases that overlapped with these conditions. Thus, a diagnosis of emphysema was made in patients who only had emphysema. We assume that this may have influenced the results.

Patients in the present study with comorbid hypertension, dyslipidemia, valvular heart disease, and cerebrovascular disease had a favorable prognosis. The association of hypertension, dyslipidemia, and cerebrovascular disease in AA patients with a favorable prognosis was reported previously. For hypertension, some reports showed a good prognosis [9], whereas others showed no prognostic involvement [23,33,34,35]. Dyslipidemia was not reported to be a prognostic factor [34]. Similarly, for cerebrovascular disease, some reports showed it to be a poor prognostic factor [32,33], and others did not [23]. However, these reports were based on surgically treated patients, and statistics were generated starting at the time of surgery. Therefore, these accumulated statistics differ from those of the present study, in which accumulation started at the time of diagnosis and included patients who did not undergo surgery. In addition, no previous study had integrated TAA and AAA as in the present study, making simple comparisons difficult. We believe that oral medications have an impact on the favorable prognosis of these diseases, and it has been reported that antihypertensive drugs, statins, and antiplatelet agents contribute to a better prognosis in patients with AA [35,36,37,38,39,40]. Among patients in the present study, 74.8% were taking an antihypertensive, 39.4% a statin, and 44.2% antiplatelet agents at and after AA diagnosis. No multivariate reports were found that evaluated valvular heart disease, making comparisons difficult.

This study has several limitations. First, it is retrospective, so some clinical and laboratory findings were unavailable. Second, our conclusions are limited by this study being a single-center review. Third, the present study is an evaluation of AA as a whole, which includes TAA, AAA, and TAAA, and the time of AA diagnosis was used as day zero. Because many other studies use the day of beginning treatment as day zero, simple comparisons are difficult to make. However, pulmonologists are often consulted about pulmonary diseases by vascular surgeons at AA diagnosis, at AA repair, and during follow-up periods. So, we decided to include all AA patients with or without AA repair, and day zero was defined as the patient’s first visit to our hospital.

## 5. Conclusions

The present study revealed that the presence of pulmonary diseases is common at the diagnosis of AA and during its course. In these patients, age and aneurysm diameter, as well as coexisting LC and IPF, were prognostic factors for mortality. When treating patients with AA, it is important to perform chest examinations, considering the possibility of coexisting pulmonary disease.

## Figures and Tables

**Table 1:** Demographics and baseline characteristics of 952 patients with TAA and/or AAA.

Characteristics	Total *n* = 952	By AA Group			
		[A] TAA *n* = 218	[B] AAA *n* = 650	[C] TAA + AAA/TAAA *n* = 84	*p*-Value
Male sex, *n* (%)	781 (82.0%)	169 (77.5%)	544 (83.7%)	68 (81.0%)	0.117
Age at AA diagnosis [years], mean (SD)	72.4 (8.4)	71.0 (9.2)	72.5 (8.2)	75.0 (7.6)	0.004
Never smoked, *n* (%)	158 (16.6%)	57 (26.1%)	90 (13.8%)	11 (13.1%)	<0.001
Shape of TAA, *n* (%)					<0.001
[1] Fusiform	168 (17.6%)	126 (57.8%)	0 (0.0%)	42 (50.0%)	
[2] Saccular	134 (14.1%)	92 (42.2%)	0 (0.0%)	42 (50.0%)	
Shape of AAA, *n* (%)					<0.001
[1] Fusiform	677 (71.1%)	0 (0.0%)	599 (92.2%)	78 (92.9%)	
[2] Saccular	57 (6.0%)	0 (0.0%)	51 (7.8%)	6 (7.1%)	
Diameter of AA (TAA or AAA, the bigger) [mm], mean (SD)	48.1 (12.9)	51 (10)	46 (13)	57 (13)	<0.001
Etiology of aneurysm, *n* (%)					0.644
[1] Atherosclerotic (degenerative)	920 (96.6%)	213 (97.7%)	626 (96.3%)	81 (96.4%)	
[2] Infectious and autoimmune (inflammatory)	30 (3.2%)	4 (1.8%)	23 (3.5%)	3 (3.6%)	
[3] Genetic (Marfan and Ehlers–Danlos)	2 (0.2%)	1 (0.5%)	1 (0.2%)	0 (0.0%)	
Pulmonary disease, *n* (%)					0.021
[1] No	424 (44.5%)	115 (52.8%)	273 (42.0%)	36 (42.9%)	
[2] Yes	528 (55.5%)	103 (47.2%)	377 (58.0%)	48 (57.1%)	
Pulmonary disease type, *n*					
[1] IPF with or without emphysema, without LC	65	7	48	10	
[2] Non-IPF ILD with or without emphysema, without LC	15	3	11	1	
[3] ILAs with or without emphysema, without LC	58	11	43	4	
[4] Emphysema without ILD, without LC	250	40	187	23	
[5] Lung cancer (concomitant)	85	26	55	4	
[6] Other lung diseases without above diseases	55	16	33	6	
IPF with or without LC, *n* (%)	84 (8.8%)	9 (4.1%)	65 (10.0%)	10 (11.9%)	0.018
ILD without LC, *n* (%)					0.143
[0] None	814 (85.5%)	197 (90.4%)	548 (84.3%)	69 (82.1%)	
[1] IPF	65 (6.8%)	7 (3.2%)	48 (7.4%)	10 (11.9%)	
[2] Non-IPF ILD	15 (1.6%)	3 (1.4%)	11 (1.7%)	1 (1.2%)	
[3] ILA	58 (6.1%)	11 (5.0%)	43 (6.6%)	4 (4.8%)	
Emphysema without ILD or LC, *n* (%)	250 (26.3%)	40 (18.3%)	187 (28.8%)	23 (27.4%)	0.010
Lung cancer, *n* (%)	85 (8.9%)	26 (11.9%)	55 (8.5%)	4 (4.8%)	0.112
Hypertension, *n* (%)	781 (82.0%)	185 (84.9%)	523 (80.5%)	73 (86.9%)	0.163
Dyslipidemia, *n* (%)	454 (47.7%)	76 (34.9%)	340 (52.3%)	38 (45.2%)	<0.001
Diabetes mellitus, *n* (%)	183 (19.2%)	38 (17.4%)	136 (20.9%)	9 (10.7%)	0.062
Coronary artery disease, *n* (%)	293 (30.8%)	37 (17.0%)	230 (35.4%)	26 (31.0%)	<0.001
Valvular heart disease, *n* (%)	111 (11.7%)	58 (26.6%)	44 (6.8%)	9 (10.7%)	<0.001
Congestive heart disease, *n* (%)	72 (7.6%)	20 (9.2%)	44 (6.8%)	8 (9.5%)	0.395
Chronic kidney disease, *n* (%)	196 (20.6%)	40 (18.3%)	132 (20.3%)	24 (28.6%)	0.137
Cerebrovascular disease, *n* (%)	171 (18.0%)	42 (19.3%)	113 (17.4%)	16 (19.0%)	0.792
Peripheral artery disease, *n* (%)	34 (3.6%)	5 (2.3%)	26 (4.0%)	3 (3.6%)	0.501
Chronic liver dysfunction, *n* (%)	24 (2.5%)	5 (2.3%)	18 (2.8%)	1 (1.2%)	0.666
History of cancer (except for LC), *n* (%)	83 (8.7%)	23 (10.6%)	51 (7.8%)	9 (10.7%)	0.375
Concomitant cancer (except for LC), *n* (%)	57 (6.0%)	8 (3.7%)	43 (6.6%)	6 (7.1%)	0.255
Outcome, Death, *n* (%)	213 (22.4%)	53 (24.3%)	143 (22.0%)	17 (20.2%)	0.689

AA = aortic aneurysm; AAA = abdominal aortic aneurysm; ILAs = interstitial lung abnormalities; ILD = interstitial lung disease; IPF = idiopathic pulmonary fibrosis; LC = lung cancer; TAA = thoracic aortic aneurysm; TAAA = thoracoabdominal aortic aneurysm.

**Table 2:** Pulmonary diseases during follow-up periods.

Pulmonary Disease	Total *n* = 126
Interstitial lung disease, *n* (%)	20 (2.1)
Acute exacerbation of idiopathic pulmonary fibrosis	12 (1.3)
Interstitial lung abnormalities	3 (0.3)
Nonspecific interstitial pneumonia	1 (0.1)
Organizing pneumonia	3 (0.3)
Others	1 (0.1)
Emphysema, *n* (%)	0 (0.0)
Infection, *n* (%)	64 (6.7)
Acute pneumonia	50 (5.3)
Non-tuberculous mycobacterial infection	4 (0.4)
Tuberculosis	3 (0.3)
Chronic pulmonary aspergillosis	2 (0.2)
Pneumocystis pneumonia	2 (0.2)
Cytomegalovirus pneumonia	1 (0.1)
Others	2 (0.2)
Lung cancer, *n* (%)	50 (5.3)
Others, *n* (%)	9 (0.9)

**Table 3:** Lung cancer at aortic aneurysm diagnosis and during follow-up periods.

Characteristics	Total *n* = 135	At AA Diagnosis *n* = 85	During Follow-Up *n* = 50	*p*-Value
Histology, *n* (%)				0.035
Adenocarcinoma	43 (31.9)	27 (31.8)	16 (32.0)	
Squamous cell carcinoma	35 (25.9)	28 (32.9)	7 (14.0)	
Other non-small cell lung carcinoma	17 (12.6)	9 (10.6)	8 (16.0)	
Small-cell carcinoma	14 (10.4)	10 (11.8)	4 (8.0)	
Only image	26 (19.3)	11 (12.9)	15 (30.0)	
Stage, *n* (%)				0.150
I	59 (43.7)	31 (36.5)	28 (56.0)	
II	21 (15.6)	16 (18.8)	5 (10.0)	
III	26 (19.3)	17 (20.0)	9 (18.0)	
IV	29 (21.5)	21 (24.7)	8 (16.0)	
1st therapy, *n* (%)				0.431
Operation	58 (43.0)	35 (41.2)	23 (46.0)	
Radiation therapy	8 (5.9)	5 (5.9)	3 (6.0)	
Chemo-radiation therapy	3 (2.2)	3 (3.5)	0 (0.0)	
Chemotherapy	33 (24.4)	24 (28.2)	9 (18.0)	
Best supportive care	32 (23.7)	17 (20.0)	15 (30.0)	
Unknown	1 (0.7)	1 (1.2)	0 (0.0)	

AA = aortic aneurysm.

**Table 4:** Causes of death.

Cause of Death	*n* = 213, *n* (%)
Aortic disease	45 (21.1)
Aortic dissection	6 (2.8)
AAA rupture	17 (8.0)
TAA rupture	20 (9.4)
Vascular graft infection	2 (0.9)
Lung cancer	59 (27.7)
Other malignancy	18 (8.5)
Cardiovascular event	20 (9.4)
Pneumonia	12 (5.6)
Other infection	8 (3.8)
Interstitial lung disease	10 (4.7)
Interstitial lung disease	4 (1.9)
AE-IPF	6 (2.8)
COPD exacerbation	1 (0.5)
Pneumothorax	1 (0.5)
Others	15 (7.0)
Unknown	24 (11.3)

AAA = abdominal aortic aneurysm; TAA = thoracic aortic aneurysm; AE = acute exacerbation; IPF = idiopathic pulmonary fibrosis; COPD = chronic obstructive pulmonary disease.

**Table 5:** Univariate and multivariate Cox regression on overall survival of 952 patients with TAA and/or AAA.

Characteristic	Univariate Model			Multivariate Model		
	HR	95% CI	*p*-Value	HR	95% CI	*p*-Value
AA group			0.625			
[A] TAA	1.00	—				
[B] AAA	0.91	0.66, 1.24				
[C] TAA + AAA/TAAA	1.14	0.66, 1.97				
Sex			0.227			
[1] Female	1.00	—				
[2] Male	1.25	0.86, 1.83				
Age group			<0.001			<0.001
[1] 27–59 years	1.00	—		1.00	—	
[2] 60–69 years	2.69	0.97, 7.46		2.27	0.81, 6.33	
[3] 70–79 years	5.50	2.02, 14.95		3.56	1.29, 9.82	
[4] 80–89 years	10.36	3.72, 28.89		6.81	2.40, 19.38	
[5] 90–94 years	24.11	6.42, 90.51		21.28	5.51, 82.25	
Smoking history			0.009			<0.001
[1] No	1.00	—		1.00	—	
[2] Yes	1.20	0.81, 1.77		1.09	0.72, 1.66	
[3] Unknown	2.29	1.35, 3.90		3.11	1.78, 5.43	
Diameter of AA (TAA or AAA, the larger)			<0.001			0.003
[1] 27–<50 mm	1.00	—		1.00	—	
[2] 50–<70 mm	1.72	1.29, 2.31		1.38	1.02, 1.85	
[3] 70–124 mm	3.20	2.04, 5.00		2.20	1.38, 3.51	
ILD without LC			<0.001			<0.001
[0] None	1.00	—		1.00	—	
[1] IPF	2.61	1.78, 3.84		3.02	1.99, 4.57	
[2] Non-IPF ILD	1.94	0.80, 4.73		1.94	0.76, 4.93	
[3] ILAs	0.88	0.47, 1.67		0.93	0.48, 1.80	
Emphysema without ILD nor LC	0.77	0.56, 1.06	0.103			
Lung cancer	5.47	4.00, 7.49	<0.001	5.85	4.11, 8.32	<0.001
Hypertension	0.40	0.30, 0.53	<0.001	0.51	0.38, 0.70	<0.001
Dyslipidemia	0.44	0.33, 0.59	<0.001	0.62	0.46, 0.84	0.002
Diabetes mellitus	0.95	0.68, 1.34	0.777			
Coronary artery disease	0.70	0.52, 0.95	0.019			
Valvular heart disease	0.65	0.40, 1.04	0.055	0.63	0.39, 1.03	0.049
Congestive heart disease	1.10	0.69, 1.74	0.696			
Chronic kidney disease	0.99	0.71, 1.38	0.949			
Cerebrovascular disease	0.73	0.50, 1.07	0.093	0.59	0.40, 0.89	0.007
Peripheral artery disease	0.50	0.19, 1.35	0.125			
Chronic liver dysfunction	1.22	0.57, 2.59	0.619			
History of cancer (except for LC)	1.58	1.01, 2.45	0.058			
Concomitant cancer (except for LC)	2.11	1.33, 3.35	0.004	1.75	1.08, 2.84	0.033

TAA = thoracic aortic aneurysm; AAA = abdominal aortic aneurysm; CI = confidence interval; HR = hazard ratio; TAAA = thoracoabdominal aortic aneurysm; LC = lung cancer; IPF = idiopathic pulmonary fibrosis; ILD = interstitial lung disease; ILAs = interstitial lung abnormalities.

## Data Availability

The data presented in this study are available from the corresponding author upon reasonable request.
